# 1,1-(Biphenyl-2,2′-diyldi­oxy)-3,3,5,5-tetra­kis­(4-bromo­methyl­phen­oxy)cyclo­triphosphazene

**DOI:** 10.1107/S1600536811016047

**Published:** 2011-05-07

**Authors:** Rui Han, Mei-Mei Chai, Jun-Liang Yang, Yong Ye

**Affiliations:** a Phosphorus Chemical Engineering Research Center of Henan Province, Department of Chemistry, Zhengzhou University, Zhengzhou 450052, People’s Republic of China

## Abstract

In the title compound, C_40_H_32_Br_4_N_3_O_6_P_3_, the cyclo­triphos­phazene ring adopts a planar conformation, with an r.m.s. deviation of 0.0247 Å. In the crystal, there is a weak inter­molecular C—H⋯O hydrogen bond as well as short inter­molecular Br⋯Br contacts [3.3352 (12) Å].

## Related literature

For general background to cyclo­triphosphazenes, see: Manners (1996) ▶. For the applications of cyclo­triphosphazene derivatives as flame retardants, see: Allcock (1977 ▶); as elastomers, see: Allcock (2000 ▶); as biomaterials, see: Trollsa & Hedrick (1998 ▶); as artificial nucleases, see: Wang, Ye, Zhong *et al.* (2009 ▶); Wang, Ye, Ju *et al.* (2009 ▶).
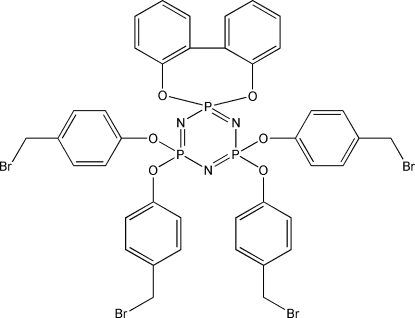


## Experimental

### 

#### Crystal data

C_40_H_32_Br_4_N_3_O_6_P_3_
*M* *_r_* = 1063.24
Monoclinic, 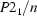

*a* = 10.991 (2) Å
*b* = 28.417 (6) Å
*c* = 14.008 (3) Å
β = 105.47 (3)°
*V* = 4216.7 (15) Å^3^
*Z* = 4
Mo *K*α radiation
μ = 3.98 mm^−1^
*T* = 293 K
0.24 × 0.20 × 0.18 mm

#### Data collection

Bruker P4 CCD diffractometer
Absorption correction: multi-scan (*SADABS*; Bruker, 2008 ▶) *T*_min_ = 0.392, *T*_max_ = 0.520
52073 measured reflections
9978 independent reflections
6049 reflections with *I* > 2σ(*I*)
*R*_int_ = 0.075

#### Refinement

*R*[*F* ^2^ > 2σ(*F* ^2^)] = 0.077
*wR*(*F* ^2^) = 0.192
*S* = 1.11
9978 reflections
505 parameters
H-atom parameters constrained
Δρ_max_ = 0.82 e Å^−3^
Δρ_min_ = −0.71 e Å^−3^

### 

Data collection: *XSCANS* (Bruker, 2008 ▶); cell refinement: *XSCANS*; data reduction: *SAINT*; program(s) used to solve structure: *SHELXS97* (Sheldrick, 2008 ▶); program(s) used to refine structure: *SHELXL97* (Sheldrick, 2008 ▶); molecular graphics: *SHELXTL* (Sheldrick, 2008 ▶); software used to prepare material for publication: *SHELXTL*.

## Supplementary Material

Crystal structure: contains datablocks global, I. DOI: 10.1107/S1600536811016047/zs2109sup1.cif

Structure factors: contains datablocks I. DOI: 10.1107/S1600536811016047/zs2109Isup2.hkl

Additional supplementary materials:  crystallographic information; 3D view; checkCIF report

## Figures and Tables

**Table 1:** Hydrogen-bond geometry (Å, °)

*D*—H⋯*A*	*D*—H	H⋯*A*	*D*⋯*A*	*D*—H⋯*A*
C33—H33*A*⋯O1^i^	0.97	2.60	3.525 (8)	160

Symmetry code: (i) 
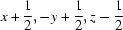
.

**Fig. 1.**
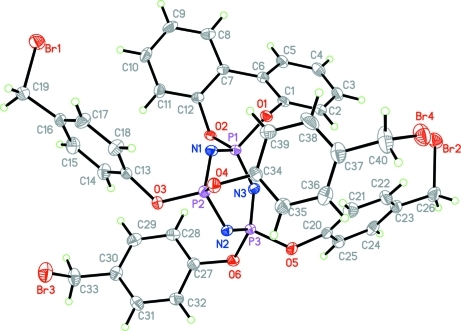
Molecular configuration and atom numbering scheme for (I), with displacement ellipsoids drawn at the 20% probability level.

## supplementary crystallographic information

### Comment

Cyclotriphosphazenes are of considerable interest not only because of their wide spectrum of chemical and physical properties, but also their importance in synthetic chemistry (Manners, 1996). Different side-group substituents affect the chemical and physical properties of the ring systems in high polymers based on a phosphazene skeleton. Various cyclotriphosphazenes have been successfully developed for a variety of applications, such as flame retardants (Allcock, 1977), elastomers (Allcock, 2000) and biomaterials (Trollsa & Hedrick, 1998), achieved by varying the nature of the substituent side group. Recently, some polydentate cyclotriphosphazene ligands were reported, which showed good nuclease activity with hydrolytic cleavage ability (Wang, Ye, Zhong *et al.*, 2009; Wang, Ye, Ju *et al.*, 2009). To obtain more insight into the selective recognization and efficient cleavage of DNA by different metal complexes of cyclotriphosphazene, the title compound, C_40_H_32_Br_4_N_3_O_6_P_3_ (I), was synthesized and its crystal structure is reported here.

In the crystal structure of (I) (Fig. 1), the part of cyclotriphosphazene ring adopts a planar conformation with an r.m.s. deviation of 0.0247 Å. There are short intermolecular Br···Br contacts: Br1···Br2^ii^ [3.3352 (12) Å] and Br3···Br4^iii^ [3.5868 (15) Å] [for symmetry codes: (ii) -*x* - 1/2, *y* - 1/2, -*z* + 1/2; (iii) -*x* + 1/2, *y* - 1/2, -*z* + 1/2]. The crystal structure is stabilized by weak intermolecular C—H···O hydrogen bond (Table 1).

### Experimental

To 30 ml of THF 2,2'-biphenyldioxy-3,3,5,5-tetrakis(4-hydroxymethylphenoxy)- cyclotriphosphazene (1.622 g, 2 mmol) was added under argon. Then, 1.1 ml of phosphorus tribromide in 10 ml of THF was added dropwise and the mixture was stirred for 5 h. The organic solvent was removed under reduced pressure and the residue was dissolved in CHCl_3_. The residue washed with aq. K_2_CO_3_ and extracted with CHCl_3_, the organic layer was dried over Na_2_SO_4_ and then evaporated under reduced pressure. The remaining residue was purified by silica gel column chromatography (CH_2_Cl_2_/PE) to provide title compound which was recrystallized from CH_2_Cl_2_ and single crystals of (I) were obtained by slow evaporation.

### Refinement

All H atoms were placed in calculated positions, with C—H = 0.93 or 0.97 Å, and included in the final cycles of refinement using a riding model, with *U*_iso_(H) = 1.2*U*_eq_(C). There is a single reflection which is considered to be affected by the beamstop.

### Crystal data

**Table:**

C_40_H_32_Br_4_N_3_O_6_P_3_	*F*(000) = 2104
*M**_r_* = 1063.24	*D*_x_ = 1.675 Mg m^−^^3^
Monoclinic, *P*2_1_/*n*	Mo *K*α radiation, λ = 0.71073 Å
Hall symbol: -P 2yn	Cell parameters from 7462 reflections
*a* = 10.991 (2) Å	θ = 2.1–27.9°
*b* = 28.417 (6) Å	µ = 3.98 mm^−^^1^
*c* = 14.008 (3) Å	*T* = 293 K
β = 105.47 (3)°	Prism, colorless
*V* = 4216.7 (15) Å^3^	0.24 × 0.20 × 0.18 mm
*Z* = 4	

### Data collection

**Table:**

Bruker P4 CCD diffractometer	9978 independent reflections
Radiation source: fine-focus sealed tube	6049 reflections with *I* > 2σ(*I*)
graphite	*R*_int_ = 0.075
ω scans	θ_max_ = 27.9°, θ_min_ = 1.7°
Absorption correction: multi-scan (*SADABS*; Bruker, 2008)	*h* = −14→14
*T*_min_ = 0.392, *T*_max_ = 0.520	*k* = −37→37
52073 measured reflections	*l* = −18→18

### Refinement

**Table:**

Refinement on *F*^2^	Primary atom site location: structure-invariant direct methods
Least-squares matrix: full	Secondary atom site location: difference Fourier map
*R*[*F*^2^ > 2σ(*F*^2^)] = 0.077	Hydrogen site location: inferred from neighbouring sites
*wR*(*F*^2^) = 0.192	H-atom parameters constrained
*S* = 1.11	*w* = 1/[σ^2^(*F*_o_^2^) + (0.0761*P*)^2^] where *P* = (*F*_o_^2^ + 2*F*_c_^2^)/3
9978 reflections	(Δ/σ)_max_ = 0.001
505 parameters	Δρ_max_ = 0.82 e Å^−^^3^
0 restraints	Δρ_min_ = −0.71 e Å^−^^3^

### Special details

**Table:**

Geometry. All e.s.d.'s (except the e.s.d. in the dihedral angle between two l.s. planes) are estimated using the full covariance matrix. The cell e.s.d.'s are taken into account individually in the estimation of e.s.d.'s in distances, angles and torsion angles; correlations between e.s.d.'s in cell parameters are only used when they are defined by crystal symmetry. An approximate (isotropic) treatment of cell e.s.d.'s is used for estimating e.s.d.'s involving l.s. planes.
Refinement. Refinement of *F*^2^ against ALL reflections. The weighted *R*-factor *wR* and goodness of fit *S* are based on *F*^2^, conventional *R*-factors *R* are based on *F*, with *F* set to zero for negative *F*^2^. The threshold expression of *F*^2^ > σ(*F*^2^) is used only for calculating *R*-factors(gt) *etc*. and is not relevant to the choice of reflections for refinement. *R*-factors based on *F*^2^ are statistically about twice as large as those based on *F*, and *R*- factors based on ALL data will be even larger.

### Fractional atomic coordinates and isotropic or equivalent isotropic displacement parameters (Å^2^)

**Table:**

	*x*	*y*	*z*	*U*_iso_*/*U*_eq_	
Br1	−0.08346 (6)	0.09289 (3)	0.36653 (6)	0.0732 (3)	
Br2	−0.10180 (6)	0.59356 (3)	0.19304 (6)	0.0773 (3)	
Br3	0.41308 (8)	0.13542 (3)	0.06958 (6)	0.0843 (3)	
Br4	0.24692 (10)	0.53274 (3)	0.55072 (7)	0.0985 (3)	
C1	−0.1461 (5)	0.3971 (2)	0.2173 (4)	0.0397 (12)	
C2	−0.1438 (5)	0.4452 (2)	0.2072 (4)	0.0478 (14)	
H2	−0.0683	0.4617	0.2283	0.057*	
C3	−0.2555 (6)	0.4686 (2)	0.1653 (5)	0.0596 (17)	
H3	−0.2547	0.5010	0.1558	0.072*	
C4	−0.3689 (6)	0.4443 (2)	0.1372 (5)	0.0605 (17)	
H4	−0.4442	0.4604	0.1114	0.073*	
C5	−0.3695 (5)	0.3956 (2)	0.1477 (4)	0.0505 (15)	
H5	−0.4457	0.3794	0.1284	0.061*	
C6	−0.2570 (5)	0.37038 (19)	0.1869 (4)	0.0405 (13)	
C7	−0.2574 (5)	0.3188 (2)	0.1952 (4)	0.0397 (12)	
C8	−0.3569 (5)	0.2945 (2)	0.2200 (4)	0.0531 (15)	
H8	−0.4232	0.3114	0.2329	0.064*	
C9	−0.3574 (6)	0.2458 (2)	0.2256 (5)	0.0616 (17)	
H9	−0.4243	0.2304	0.2410	0.074*	
C10	−0.2592 (6)	0.2202 (2)	0.2083 (5)	0.0599 (17)	
H10	−0.2593	0.1876	0.2134	0.072*	
C11	−0.1597 (5)	0.2428 (2)	0.1833 (4)	0.0514 (15)	
H11	−0.0936	0.2255	0.1713	0.062*	
C12	−0.1602 (5)	0.2911 (2)	0.1766 (4)	0.0408 (13)	
C13	0.2596 (5)	0.21640 (18)	0.3066 (4)	0.0394 (12)	
C14	0.1915 (6)	0.1900 (2)	0.2275 (4)	0.0555 (16)	
H14	0.1855	0.1997	0.1630	0.067*	
C15	0.1329 (6)	0.1493 (2)	0.2442 (5)	0.0600 (17)	
H15	0.0845	0.1322	0.1910	0.072*	
C16	0.1454 (5)	0.13370 (19)	0.3407 (5)	0.0506 (15)	
C17	0.2122 (6)	0.1612 (2)	0.4168 (5)	0.0582 (16)	
H17	0.2182	0.1518	0.4815	0.070*	
C18	0.2703 (6)	0.2019 (2)	0.4018 (4)	0.0551 (16)	
H18	0.3163	0.2195	0.4552	0.066*	
C19	0.0912 (6)	0.0870 (2)	0.3589 (6)	0.069 (2)	
H19A	0.1424	0.0739	0.4204	0.082*	
H19B	0.0942	0.0655	0.3058	0.082*	
C20	0.2409 (5)	0.46520 (19)	0.1584 (4)	0.0443 (14)	
C21	0.2325 (6)	0.4852 (2)	0.2471 (5)	0.0619 (17)	
H21	0.2668	0.4699	0.3069	0.074*	
C22	0.1733 (6)	0.5276 (2)	0.2451 (5)	0.0606 (17)	
H22	0.1700	0.5415	0.3044	0.073*	
C23	0.1184 (5)	0.5500 (2)	0.1571 (6)	0.0575 (17)	
C24	0.1274 (5)	0.5302 (2)	0.0719 (5)	0.0545 (16)	
H24	0.0915	0.5455	0.0123	0.065*	
C25	0.1886 (5)	0.4877 (2)	0.0705 (4)	0.0505 (15)	
H25	0.1943	0.4747	0.0109	0.061*	
C26	0.0582 (6)	0.5975 (2)	0.1576 (7)	0.086 (3)	
H26A	0.0436	0.6116	0.0924	0.104*	
H26B	0.1156	0.6178	0.2045	0.104*	
C27	0.2824 (5)	0.3118 (2)	0.0172 (4)	0.0453 (13)	
C28	0.1792 (6)	0.2826 (2)	0.0044 (4)	0.0545 (15)	
H28	0.1028	0.2937	0.0120	0.065*	
C29	0.1932 (6)	0.2355 (2)	−0.0206 (4)	0.0580 (16)	
H29	0.1246	0.2153	−0.0299	0.070*	
C30	0.3068 (6)	0.2184 (2)	−0.0318 (4)	0.0518 (15)	
C31	0.4050 (6)	0.2488 (2)	−0.0236 (5)	0.0591 (16)	
H31	0.4797	0.2380	−0.0351	0.071*	
C32	0.3950 (5)	0.2958 (2)	0.0020 (4)	0.0538 (15)	
H32	0.4630	0.3162	0.0088	0.065*	
C33	0.3209 (7)	0.1675 (2)	−0.0525 (5)	0.0693 (19)	
H33A	0.3666	0.1641	−0.1025	0.083*	
H33B	0.2383	0.1533	−0.0775	0.083*	
C34	0.3556 (5)	0.3579 (2)	0.4642 (4)	0.0453 (14)	
C35	0.4494 (6)	0.3902 (2)	0.4700 (5)	0.0605 (17)	
H35	0.5128	0.3846	0.4388	0.073*	
C36	0.4494 (7)	0.4315 (2)	0.5229 (5)	0.0670 (18)	
H36	0.5131	0.4536	0.5274	0.080*	
C37	0.3557 (8)	0.4398 (3)	0.5684 (5)	0.073 (2)	
C38	0.2603 (8)	0.4073 (3)	0.5598 (5)	0.081 (2)	
H38	0.1958	0.4134	0.5896	0.097*	
C39	0.2586 (6)	0.3658 (2)	0.5074 (5)	0.0678 (19)	
H39	0.1939	0.3440	0.5017	0.081*	
C40	0.3541 (10)	0.4844 (3)	0.6263 (6)	0.104 (3)	
H40A	0.4395	0.4964	0.6490	0.125*	
H40B	0.3253	0.4771	0.6843	0.125*	
N1	0.1362 (4)	0.31181 (15)	0.2933 (3)	0.0398 (10)	
N2	0.3402 (4)	0.34123 (15)	0.2342 (3)	0.0408 (10)	
N3	0.1042 (4)	0.37315 (15)	0.1405 (3)	0.0410 (11)	
O1	−0.0320 (3)	0.37457 (12)	0.2693 (3)	0.0400 (9)	
O2	−0.0652 (3)	0.31282 (12)	0.1399 (2)	0.0386 (8)	
O3	0.3244 (3)	0.25632 (12)	0.2862 (3)	0.0473 (9)	
O4	0.3618 (3)	0.31523 (13)	0.4151 (3)	0.0467 (9)	
O5	0.3091 (4)	0.42359 (13)	0.1597 (4)	0.0642 (12)	
O6	0.2786 (4)	0.35924 (13)	0.0467 (3)	0.0504 (10)	
P1	0.04465 (12)	0.34309 (5)	0.21146 (10)	0.0365 (3)	
P2	0.28306 (12)	0.30824 (5)	0.30222 (11)	0.0394 (3)	
P3	0.25256 (13)	0.37218 (5)	0.14930 (11)	0.0419 (4)	

### Atomic displacement parameters (Å^2^)

**Table:**

	*U*^11^	*U*^22^	*U*^33^	*U*^12^	*U*^13^	*U*^23^
Br1	0.0496 (4)	0.0666 (5)	0.1086 (6)	−0.0111 (3)	0.0299 (4)	−0.0175 (4)
Br2	0.0483 (4)	0.0767 (5)	0.1062 (6)	0.0045 (3)	0.0193 (4)	−0.0218 (4)
Br3	0.1166 (7)	0.0600 (5)	0.0737 (5)	0.0086 (4)	0.0206 (5)	0.0056 (4)
Br4	0.1407 (8)	0.0686 (5)	0.0916 (6)	0.0241 (5)	0.0405 (6)	0.0054 (4)
C1	0.037 (3)	0.048 (3)	0.038 (3)	0.001 (2)	0.015 (2)	−0.009 (2)
C2	0.047 (3)	0.043 (3)	0.060 (4)	−0.004 (3)	0.026 (3)	−0.008 (3)
C3	0.081 (5)	0.044 (4)	0.063 (4)	0.013 (3)	0.034 (4)	0.001 (3)
C4	0.051 (4)	0.063 (4)	0.068 (4)	0.020 (3)	0.016 (3)	−0.001 (3)
C5	0.045 (3)	0.052 (4)	0.055 (4)	0.005 (3)	0.014 (3)	−0.008 (3)
C6	0.039 (3)	0.046 (3)	0.038 (3)	0.005 (2)	0.011 (2)	0.004 (2)
C7	0.032 (3)	0.047 (3)	0.040 (3)	−0.003 (2)	0.011 (2)	0.001 (2)
C8	0.044 (3)	0.069 (4)	0.048 (4)	−0.008 (3)	0.015 (3)	−0.001 (3)
C9	0.060 (4)	0.065 (4)	0.064 (4)	−0.031 (4)	0.024 (3)	−0.002 (3)
C10	0.080 (5)	0.039 (3)	0.060 (4)	−0.018 (3)	0.018 (3)	−0.001 (3)
C11	0.051 (3)	0.039 (3)	0.061 (4)	−0.004 (3)	0.011 (3)	−0.005 (3)
C12	0.031 (3)	0.049 (3)	0.041 (3)	−0.004 (2)	0.007 (2)	0.000 (2)
C13	0.035 (3)	0.036 (3)	0.049 (3)	0.004 (2)	0.014 (2)	−0.002 (2)
C14	0.073 (4)	0.048 (4)	0.041 (4)	0.001 (3)	0.008 (3)	0.004 (3)
C15	0.063 (4)	0.052 (4)	0.056 (4)	−0.007 (3)	0.001 (3)	−0.012 (3)
C16	0.039 (3)	0.036 (3)	0.079 (5)	0.003 (3)	0.020 (3)	0.000 (3)
C17	0.075 (4)	0.057 (4)	0.045 (4)	−0.013 (3)	0.020 (3)	0.005 (3)
C18	0.066 (4)	0.056 (4)	0.039 (4)	−0.013 (3)	0.007 (3)	−0.001 (3)
C19	0.063 (4)	0.048 (4)	0.107 (6)	−0.003 (3)	0.045 (4)	0.001 (4)
C20	0.038 (3)	0.032 (3)	0.066 (4)	−0.001 (2)	0.020 (3)	0.004 (3)
C21	0.075 (4)	0.063 (4)	0.050 (4)	−0.001 (4)	0.019 (3)	0.011 (3)
C22	0.064 (4)	0.054 (4)	0.067 (4)	−0.001 (3)	0.023 (3)	−0.019 (3)
C23	0.044 (3)	0.043 (3)	0.089 (5)	−0.004 (3)	0.024 (3)	−0.008 (4)
C24	0.050 (4)	0.044 (3)	0.062 (4)	−0.007 (3)	0.001 (3)	0.013 (3)
C25	0.056 (4)	0.052 (4)	0.045 (4)	−0.008 (3)	0.016 (3)	−0.009 (3)
C26	0.061 (4)	0.047 (4)	0.161 (8)	0.000 (3)	0.046 (5)	0.000 (5)
C27	0.052 (3)	0.047 (3)	0.038 (3)	0.002 (3)	0.015 (3)	0.006 (2)
C28	0.051 (4)	0.064 (4)	0.052 (4)	−0.003 (3)	0.020 (3)	0.001 (3)
C29	0.059 (4)	0.066 (4)	0.049 (4)	−0.014 (3)	0.014 (3)	0.000 (3)
C30	0.062 (4)	0.049 (4)	0.041 (3)	−0.006 (3)	0.008 (3)	−0.005 (3)
C31	0.065 (4)	0.058 (4)	0.057 (4)	0.012 (3)	0.022 (3)	−0.006 (3)
C32	0.041 (3)	0.062 (4)	0.057 (4)	−0.001 (3)	0.013 (3)	0.003 (3)
C33	0.101 (5)	0.049 (4)	0.054 (4)	0.008 (4)	0.014 (4)	−0.006 (3)
C34	0.047 (3)	0.047 (3)	0.040 (3)	0.005 (3)	0.008 (3)	−0.005 (3)
C35	0.059 (4)	0.059 (4)	0.063 (4)	−0.004 (3)	0.015 (3)	−0.002 (3)
C36	0.076 (5)	0.051 (4)	0.070 (5)	−0.004 (4)	0.012 (4)	0.007 (3)
C37	0.099 (6)	0.056 (4)	0.059 (4)	0.006 (4)	0.012 (4)	−0.005 (3)
C38	0.103 (6)	0.088 (6)	0.065 (5)	0.017 (5)	0.046 (4)	−0.016 (4)
C39	0.069 (4)	0.068 (5)	0.076 (5)	−0.013 (4)	0.035 (4)	−0.012 (4)
C40	0.181 (9)	0.056 (5)	0.068 (5)	0.022 (5)	0.022 (5)	−0.005 (4)
N1	0.033 (2)	0.042 (3)	0.047 (3)	0.0026 (19)	0.016 (2)	0.004 (2)
N2	0.031 (2)	0.035 (2)	0.059 (3)	−0.0023 (19)	0.018 (2)	0.002 (2)
N3	0.034 (2)	0.037 (2)	0.053 (3)	0.0041 (19)	0.014 (2)	0.010 (2)
O1	0.0313 (19)	0.043 (2)	0.047 (2)	0.0017 (16)	0.0130 (16)	−0.0061 (17)
O2	0.0345 (18)	0.043 (2)	0.042 (2)	−0.0042 (16)	0.0176 (16)	−0.0089 (16)
O3	0.048 (2)	0.032 (2)	0.067 (3)	0.0024 (17)	0.0243 (19)	−0.0022 (18)
O4	0.041 (2)	0.043 (2)	0.052 (2)	0.0017 (17)	0.0053 (18)	−0.0025 (18)
O5	0.045 (2)	0.032 (2)	0.125 (4)	0.0020 (18)	0.040 (2)	0.004 (2)
O6	0.062 (2)	0.044 (2)	0.057 (2)	0.0070 (19)	0.036 (2)	0.0122 (19)
P1	0.0320 (7)	0.0372 (7)	0.0431 (8)	−0.0004 (6)	0.0148 (6)	−0.0016 (6)
P2	0.0346 (7)	0.0346 (7)	0.0494 (9)	0.0015 (6)	0.0117 (6)	−0.0005 (6)
P3	0.0394 (8)	0.0330 (7)	0.0592 (10)	0.0005 (6)	0.0236 (7)	0.0036 (6)

### Geometric parameters (Å, °)

**Table:**

Br1—C19	1.958 (6)	C23—C24	1.347 (9)
Br2—C26	1.953 (7)	C23—C26	1.503 (8)
Br3—C33	1.963 (6)	C24—C25	1.385 (8)
Br4—C40	1.932 (8)	C24—H24	0.9300
C1—C2	1.374 (7)	C25—H25	0.9300
C1—C6	1.403 (7)	C26—H26A	0.9700
C1—O1	1.423 (6)	C26—H26B	0.9700
C2—C3	1.382 (8)	C27—C28	1.379 (8)
C2—H2	0.9300	C27—C32	1.387 (8)
C3—C4	1.388 (9)	C27—O6	1.413 (7)
C3—H3	0.9300	C28—C29	1.401 (9)
C4—C5	1.392 (8)	C28—H28	0.9300
C4—H4	0.9300	C29—C30	1.387 (8)
C5—C6	1.407 (7)	C29—H29	0.9300
C5—H5	0.9300	C30—C31	1.363 (8)
C6—C7	1.470 (7)	C30—C33	1.492 (8)
C7—C12	1.407 (7)	C31—C32	1.397 (8)
C7—C8	1.414 (7)	C31—H31	0.9300
C8—C9	1.385 (8)	C32—H32	0.9300
C8—H8	0.9300	C33—H33A	0.9700
C9—C10	1.375 (9)	C33—H33B	0.9700
C9—H9	0.9300	C34—C35	1.367 (8)
C10—C11	1.391 (8)	C34—C39	1.378 (8)
C10—H10	0.9300	C34—O4	1.405 (6)
C11—C12	1.374 (7)	C35—C36	1.389 (9)
C11—H11	0.9300	C35—H35	0.9300
C12—O2	1.422 (6)	C36—C37	1.368 (10)
C13—C18	1.370 (8)	C36—H36	0.9300
C13—C14	1.382 (7)	C37—C38	1.377 (10)
C13—O3	1.409 (6)	C37—C40	1.507 (10)
C14—C15	1.373 (8)	C38—C39	1.386 (9)
C14—H14	0.9300	C38—H38	0.9300
C15—C16	1.393 (9)	C39—H39	0.9300
C15—H15	0.9300	C40—H40A	0.9700
C16—C17	1.368 (8)	C40—H40B	0.9700
C16—C19	1.503 (8)	N1—P1	1.581 (4)
C17—C18	1.364 (8)	N1—P2	1.589 (4)
C17—H17	0.9300	N2—P2	1.581 (4)
C18—H18	0.9300	N2—P3	1.583 (4)
C19—H19A	0.9700	N3—P1	1.579 (4)
C19—H19B	0.9700	N3—P3	1.602 (4)
C20—C25	1.371 (8)	O1—P1	1.591 (4)
C20—C21	1.390 (8)	O2—P1	1.599 (4)
C20—O5	1.398 (6)	O3—P2	1.577 (4)
C21—C22	1.366 (9)	O4—P2	1.600 (4)
C21—H21	0.9300	O5—P3	1.579 (4)
C22—C23	1.377 (9)	O6—P3	1.582 (4)
C22—H22	0.9300		
			
C2—C1—C6	123.1 (5)	Br2—C26—H26A	109.5
C2—C1—O1	117.6 (5)	C23—C26—H26B	109.5
C6—C1—O1	119.1 (5)	Br2—C26—H26B	109.5
C1—C2—C3	118.9 (5)	H26A—C26—H26B	108.5
C1—C2—H2	120.0	C28—C27—C32	121.3 (6)
C3—C2—H2	120.0	C28—C27—O6	121.7 (5)
C2—C3—C4	120.6 (6)	C32—C27—O6	117.0 (5)
C4—C3—H3	120.0	C27—C28—C29	117.8 (6)
C2—C3—H3	120.0	C27—C28—H28	120.0
C3—C4—C5	119.7 (6)	C29—C28—H28	120.0
C3—C4—H4	120.0	C30—C29—C28	121.7 (6)
C5—C4—H4	120.0	C30—C29—H29	120.0
C4—C5—C6	121.2 (5)	C28—C29—H29	120.0
C4—C5—H5	120.0	C31—C30—C29	119.0 (6)
C6—C5—H5	120.0	C31—C30—C33	120.6 (6)
C1—C6—C5	116.4 (5)	C29—C30—C33	120.4 (6)
C1—C6—C7	122.5 (4)	C30—C31—C32	120.8 (6)
C5—C6—C7	121.1 (5)	C30—C31—H31	120.0
C12—C7—C8	116.5 (5)	C32—C31—H31	120.0
C12—C7—C6	121.7 (5)	C27—C32—C31	119.2 (6)
C8—C7—C6	121.8 (5)	C27—C32—H32	120.0
C9—C8—C7	121.1 (6)	C31—C32—H32	120.0
C9—C8—H8	120.0	C30—C33—Br3	109.7 (4)
C7—C8—H8	120.0	C30—C33—H33A	109.5
C10—C9—C8	120.2 (6)	Br3—C33—H33A	109.5
C10—C9—H9	120.0	C30—C33—H33B	109.5
C8—C9—H9	120.0	Br3—C33—H33B	109.5
C9—C10—C11	120.4 (6)	H33A—C33—H33B	108.5
C9—C10—H10	120.0	C35—C34—C39	121.6 (6)
C11—C10—H10	120.0	C35—C34—O4	118.3 (5)
C12—C11—C10	119.3 (6)	C39—C34—O4	120.1 (5)
C12—C11—H11	120.0	C34—C35—C36	119.5 (6)
C10—C11—H11	120.0	C34—C35—H35	120.0
C11—C12—C7	122.4 (5)	C36—C35—H35	120.0
C11—C12—O2	117.9 (5)	C37—C36—C35	120.1 (7)
C7—C12—O2	119.4 (5)	C37—C36—H36	120.0
C18—C13—C14	120.3 (5)	C35—C36—H36	120.0
C18—C13—O3	121.5 (5)	C36—C37—C38	119.6 (7)
C14—C13—O3	118.0 (5)	C36—C37—C40	120.9 (8)
C15—C14—C13	119.9 (6)	C38—C37—C40	119.5 (8)
C15—C14—H14	120.0	C37—C38—C39	121.2 (7)
C13—C14—H14	120.0	C37—C38—H38	120.0
C14—C15—C16	120.3 (6)	C39—C38—H38	120.0
C14—C15—H15	120.0	C34—C39—C38	118.0 (7)
C16—C15—H15	120.0	C34—C39—H39	120.0
C17—C16—C15	117.9 (5)	C38—C39—H39	120.0
C17—C16—C19	121.8 (6)	C37—C40—Br4	113.3 (5)
C15—C16—C19	120.3 (6)	C37—C40—H40A	109.5
C18—C17—C16	122.7 (6)	Br4—C40—H40A	109.5
C16—C17—H17	120.0	C37—C40—H40B	109.5
C18—C17—H17	120.0	Br4—C40—H40B	109.5
C17—C18—C13	118.9 (5)	H40A—C40—H40B	108.5
C13—C18—H18	120.0	P1—N1—P2	122.0 (3)
C17—C18—H18	120.0	P2—N2—P3	121.5 (2)
C16—C19—Br1	111.9 (4)	P1—N3—P3	121.8 (3)
C16—C19—H19A	109.5	C1—O1—P1	120.4 (3)
Br1—C19—H19A	109.5	C12—O2—P1	120.8 (3)
C16—C19—H19B	109.5	C13—O3—P2	123.0 (3)
Br1—C19—H19B	109.5	C34—O4—P2	120.5 (3)
H19A—C19—H19B	108.5	C20—O5—P3	125.8 (3)
C25—C20—C21	120.0 (5)	C27—O6—P3	120.9 (3)
C25—C20—O5	120.1 (5)	N3—P1—N1	118.0 (2)
C21—C20—O5	119.8 (5)	N3—P1—O1	112.4 (2)
C22—C21—C20	119.1 (6)	N1—P1—O1	105.5 (2)
C22—C21—H21	120.0	N3—P1—O2	105.5 (2)
C20—C21—H21	120.0	N1—P1—O2	112.3 (2)
C21—C22—C23	121.3 (6)	O1—P1—O2	101.99 (18)
C21—C22—H22	120.0	O3—P2—N2	107.0 (2)
C23—C22—H22	120.0	O3—P2—N1	111.9 (2)
C24—C23—C22	118.8 (6)	N2—P2—N1	118.2 (2)
C24—C23—C26	121.0 (7)	O3—P2—O4	99.0 (2)
C22—C23—C26	120.0 (7)	N2—P2—O4	108.9 (2)
C23—C24—C25	121.8 (6)	N1—P2—O4	109.9 (2)
C23—C24—H24	120.0	O5—P3—O6	97.9 (2)
C25—C24—H24	120.0	O5—P3—N2	107.4 (2)
C20—C25—C24	118.9 (6)	O6—P3—N2	109.4 (2)
C20—C25—H25	120.0	O5—P3—N3	111.0 (2)
C24—C25—H25	120.0	O6—P3—N3	111.2 (2)
C23—C26—Br2	112.2 (5)	N2—P3—N3	118.1 (2)
C23—C26—H26A	109.5		
			
O2—P1—O1—C1	−44.8 (4)	C5—C6—C7—C8	−36.7 (8)
N1—P1—O1—C1	−162.3 (4)	C1—C6—C7—C8	143.2 (6)
N3—P1—O1—C1	67.8 (4)	C6—C7—C12—O2	−6.0 (8)
O1—P1—O2—C12	−45.8 (4)	C12—C7—C8—C9	0.1 (8)
N1—P1—O2—C12	66.8 (4)	C8—C7—C12—C11	−0.9 (8)
N3—P1—O2—C12	−163.4 (4)	C6—C7—C12—C11	−179.5 (5)
N3—P1—N1—P2	−2.8 (4)	C8—C7—C12—O2	172.6 (5)
N1—P1—N3—P3	0.5 (4)	C6—C7—C8—C9	178.7 (5)
O1—P1—N3—P3	123.7 (3)	C7—C8—C9—C10	0.9 (9)
O2—P1—N3—P3	−126.0 (3)	C8—C9—C10—C11	−1.2 (10)
O1—P1—N1—P2	−129.4 (3)	C9—C10—C11—C12	0.4 (9)
O2—P1—N1—P2	120.2 (3)	C10—C11—C12—C7	0.7 (9)
N1—P2—O3—C13	−26.9 (5)	C10—C11—C12—O2	−172.9 (5)
O4—P2—O3—C13	88.9 (4)	O3—C13—C14—C15	176.3 (5)
O3—P2—N1—P1	−118.6 (3)	C18—C13—C14—C15	0.8 (9)
N2—P2—O3—C13	−158.0 (4)	O3—C13—C18—C17	−175.6 (5)
O3—P2—O4—C34	179.8 (4)	C14—C13—C18—C17	−0.3 (9)
N1—P2—O4—C34	−62.8 (4)	C13—C14—C15—C16	−2.2 (10)
N2—P2—O4—C34	68.2 (4)	C14—C15—C16—C19	−174.5 (6)
N1—P2—N2—P3	−8.1 (4)	C14—C15—C16—C17	3.0 (9)
N2—P2—N1—P1	6.5 (4)	C15—C16—C17—C18	−2.5 (10)
O3—P2—N2—P3	119.4 (3)	C19—C16—C17—C18	174.9 (6)
O4—P2—N1—P1	132.5 (3)	C15—C16—C19—Br1	−89.6 (7)
O4—P2—N2—P3	−134.4 (3)	C17—C16—C19—Br1	93.1 (7)
N2—P3—O5—C20	−132.4 (5)	C16—C17—C18—C13	1.2 (10)
N3—P3—O5—C20	−2.0 (6)	O5—C20—C21—C22	175.3 (6)
O6—P3—O5—C20	114.4 (5)	O5—C20—C25—C24	−176.5 (5)
O5—P3—N2—P2	132.2 (3)	C21—C20—C25—C24	−0.3 (9)
O6—P3—N2—P2	−122.6 (3)	C25—C20—C21—C22	−0.9 (9)
N3—P3—N2—P2	5.8 (4)	C20—C21—C22—C23	2.2 (10)
O5—P3—O6—C27	159.7 (4)	C21—C22—C23—C24	−2.2 (10)
N2—P3—O6—C27	48.1 (5)	C21—C22—C23—C26	−177.7 (6)
N3—P3—O6—C27	−84.1 (5)	C22—C23—C24—C25	1.0 (9)
O5—P3—N3—P1	−126.6 (3)	C22—C23—C26—Br2	−73.1 (7)
O6—P3—N3—P1	125.6 (3)	C24—C23—C26—Br2	111.5 (6)
N2—P3—N3—P1	−2.0 (4)	C26—C23—C24—C25	176.4 (6)
P1—O1—C1—C2	−108.8 (5)	C23—C24—C25—C20	0.3 (9)
P1—O1—C1—C6	76.9 (6)	C32—C27—C28—C29	2.6 (8)
P1—O2—C12—C7	75.7 (6)	O6—C27—C28—C29	−177.2 (5)
P1—O2—C12—C11	−110.5 (5)	O6—C27—C32—C31	177.7 (5)
P2—O3—C13—C18	−74.7 (6)	C28—C27—C32—C31	−2.1 (8)
P2—O3—C13—C14	109.9 (5)	C27—C28—C29—C30	0.3 (8)
P2—O4—C34—C39	84.1 (6)	C28—C29—C30—C33	176.4 (5)
P2—O4—C34—C35	−97.7 (6)	C28—C29—C30—C31	−3.7 (8)
P3—O5—C20—C21	94.2 (7)	C33—C30—C31—C32	−175.9 (6)
P3—O5—C20—C25	−89.6 (7)	C29—C30—C31—C32	4.1 (9)
P3—O6—C27—C32	−118.7 (5)	C29—C30—C33—Br3	−101.7 (6)
P3—O6—C27—C28	61.1 (7)	C31—C30—C33—Br3	78.3 (7)
O1—C1—C6—C5	171.9 (5)	C30—C31—C32—C27	−1.3 (9)
C6—C1—C2—C3	0.1 (9)	O4—C34—C35—C36	−176.4 (5)
O1—C1—C2—C3	−174.1 (5)	C39—C34—C35—C36	1.8 (10)
O1—C1—C6—C7	−8.0 (8)	O4—C34—C39—C38	176.4 (6)
C2—C1—C6—C5	−2.2 (8)	C35—C34—C39—C38	−1.7 (9)
C2—C1—C6—C7	178.0 (5)	C34—C35—C36—C37	−0.3 (10)
C1—C2—C3—C4	2.4 (9)	C35—C36—C37—C38	−1.2 (11)
C2—C3—C4—C5	−2.6 (10)	C35—C36—C37—C40	179.9 (7)
C3—C4—C5—C6	0.3 (9)	C36—C37—C38—C39	1.3 (11)
C4—C5—C6—C7	−178.2 (5)	C40—C37—C38—C39	−179.8 (7)
C4—C5—C6—C1	1.9 (8)	C36—C37—C40—Br4	96.5 (9)
C1—C6—C7—C12	−38.3 (8)	C38—C37—C40—Br4	−82.5 (8)
C5—C6—C7—C12	141.9 (6)	C37—C38—C39—C34	0.2 (10)

### Hydrogen-bond geometry (Å, °)

**Table:**

*D*—H···*A*	*D*—H	H···*A*	*D*···*A*	*D*—H···*A*
C33—H33A···O1^i^	0.97	2.60	3.525 (8)	160

Symmetry codes: (i) *x*+1/2, −*y*+1/2, *z*−1/2.
